# Lead Pipe Poison

**Published:** 1871-11

**Authors:** 


// HALL'S

### JOURNAL OF HEALTH.

VOL. XVIII. — NOVEMBER, 1871. — No. XI.

### LEAD PIPE POISON.

A GREAT deal has been written about the poisonous nature of water brought into our houses by lead pipes. There is but very little truth in it. If our water is poisoned by the decomposition of the lead, why are not the lead pipes eaten up before now, and why is not everybody on Manhattan Island removed before now to Greenwood ?

There is nothing so good to impress an important truth as a story with a fact, so we tell it at our own expense.

A Dutch chemist came to us one day, and said, " I can make white lead in twenty-four hours at a trifling cost; the common plan requires from three to six months; here is a wonderful saving of capital, for ten thousand dollars can be turned ten times in ten months, instead of twice." The idea was to present a large surface of lead to a corrosive one of the strong acids for a few hours; take it out; as soon as dried, the whole surface is covered with pure white lead; wash it off with water, dry it, and the whole work is done, at about a thousand per cent. profit. We turned it over to Professor Mitchell, the matchless Astronomical Lecturer, for examination. Every experiment corroborated the first.

All that was to be done was to keep on applying the acid to very fine lead wire, wash off the white lead, and repeat the operation until the lead disappeared. What visions of millions pouring into our coffers every year! Lead works were erected over the river, without the slightest doubt whatever of a triumphant success, and we were going to establish white lead factories all the way from the Jersey Land Flats to the capital of the great Mogul. We were going to banish slavery from the universe by catching every nigger at night, painting him white, and then letting him go. We started the factory, — made ever so much white lead the first day. The Professor had a wonderful mind for notes, for accuracy; the reader will remember how he told us in the Academy of Music, where five thousand auditors listened with rapture to lectures which lasted for hours, the only instrument for measuring all worlds, and explaining their complicated motions, being the little rod, a few feet long, which he held in his hand, and his own magnificent word painting, — as we were saying, he told us of an instrument he had made for measuring space and time to the millionth part of a second of time or inch of space; so he measured or rather weighed the white lead the first day, and reported very satisfactory results. The next day's operations were not quite so encouraging. In less than a week there was no white lead at all; no money was made, no niggers were transmogrified into white people, slavery was not abolished, the war came on, and "I alone am left to tell the tale!"

But why did hair-fine lead wire cease to corrode and yield white lead? Because in the process of decomposition a film was gradually formed, which no water could wash away; not even aquafortis could dissolve or penetrate it, so impervious was it. Nothing known could remove it but fire, and to melt the lead over again at each chemical washing left so much refuse dross, and the expense of converting the remnant into hair-fine wire was so great, that a continuance of the operation was ruinous.

Water will indeed corrode lead pipes to a slight extent for a short time, but the process of corrosion soon forms an impenetrable film, and the pipe becomes innocuous unless this film is broken, or is scaled off in parts; but even then it will form again. Hence we read with pleasure in the "Galaxy" for October, 1871, page 568, "Dr. Lissaur states that lead pipes for conducting water, during the first four or five weeks of their use, contaminate such water with lead, but afterward, a strongly adhering coating of oxide of lead is formed on the inside of the pipes, when no further danger need be apprehended. As soon as water contains about four and a half grains per gallon, kept in solution by carbonic acid, pipes are not acted upon." That is, if there is a very small amount of lead in the water, the water ceases to dissolve the lead further. But as science often leads us by the nose until we get into a muddle and then leaves us, in the best way we can, to find our way back to solid land, it will be safer, at least for the present, to let our water pipes run a minute before we take water to drink, or use for cooking in the morning; and in going into a new house, or into one in which water pipes have been just placed, it is better not to use the water for drinking or cooking for at least one month. And in returning home after some weeks' absence, in summer excursions or for other reasons, take the same precautions. Perhaps in these things we find the reason of some persons sickening and dying soon after getting into new houses.

